# Risk of spontaneous pneumothorax due to air travel and diving in patients with Birt–Hogg–Dubé syndrome

**DOI:** 10.1186/s40064-016-3009-4

**Published:** 2016-09-07

**Authors:** P. C. Johannesma, I. van de Beek, J. W. T. van der Wel, M. A. Paul, A. C. Houweling, M. A. Jonker, J. H. T. M. van Waesberghe, R. Reinhard, Th. M. Starink, R. J. A. van Moorselaar, F. H. Menko, P. E. Postmus

**Affiliations:** 1 Department of Pulmonary Diseases, VU University Medical Center, PO Box 7057, 1007 MB Amsterdam, The Netherlands; 2 Department of Clinical Genetics, VU University Medical Center, Amsterdam, The Netherlands; 3 Department of Thoracic Surgery, VU University Medical Center, Amsterdam, The Netherlands; 4 Department of Epidemiology and Biostatistics, VU University Medical Center, Amsterdam, The Netherlands; 5 Department of Radiology, VU University Medical Center, Amsterdam, The Netherlands; 6 Department of Dermatology, VU University Medical Center, Amsterdam, The Netherlands; 7 Department of Urology, VU University Medical Center, Amsterdam, The Netherlands; 8 Family Cancer Clinic, Netherlands Cancer Institute, Amsterdam, The Netherlands; 9 Clatterbridge Cancer Centre, Liverpool Heart and Chest Hospital, Liverpool, UK; 10 Department of Thoracic Oncology, University of Liverpool, Liverpool, UK

**Keywords:** Air travel, Diving, Spontaneous pneumothorax, Lung cysts, Birt–Hogg–Dubé syndrome, Folliculin, *FLCN*, Renal cell cancer, Thoracic computed tomography

## Abstract

**Background and objectives:**

Birt–Hogg–Dubé syndrome is an autosomal dominant disorder characterized by skin fibrofolliculomas, lung cysts, spontaneous pneumothorax and renal cell cancer due to germline folliculin (FLCN) mutations (Menko et al. in Lancet Oncol 10(12):1199–1206, 2009). The aim of this study was to evaluate the incidence of spontaneous pneumothorax in patients with BHD during or shortly after air travel and diving.

**Methods:**

A questionnaire was sent to a cohort of 190 BHD patients and the medical files of these patients were evaluated. The diagnosis of BHD was confirmed by *FLCN* mutations analysis in all patients. We assessed how many spontaneous pneumothoraces (SP) occurred within 1 month after air travel or diving.

**Results:**

In total 158 (83.2 %) patients returned the completed questionnaire. A total of 145 patients had a history of air travel. Sixty-one of them had a history of SP (42.1 %), with a mean of 2.48 episodes (range 1–10). Twenty-four (35.8 %) patients had a history of pneumothorax on both sides. Thirteen patients developed SP < 1 month after air travel (9.0 %) and two patients developed a SP < 1 month after diving (3.7 %). We found in this population of BHD patients a pneumothorax risk of 0.63 % per flight and a risk of 0.33 % per episode of diving. Symptoms possible related to SP were perceived in 30 patients (20.7 %) after air travel, respectively in ten patients (18.5 %) after diving.

**Conclusion:**

Based on the results presented in this retrospective study, exposure of BHD patients to considerable changes in atmospheric pressure associated with flying and diving may be related to an increased risk for developing a symptomatic pneumothorax. Symptoms reported during or shortly after flying and diving might be related to the early phase of pneumothorax. An individualized advice should be given, taking also into account patients’ preferences and needs.

## Background

Changes in atmospheric pressure are related to a higher incidence of spontaneous primary pneumothorax. Atmospheric pressure changes from day to day are usually small. However, during flying or diving significant changes in pressure will occur. Therefore it might be expected that patients with a predisposition for air trapping, such as cystic lesions not connected to the airways, have a considerable risk of developing a pneumothorax. Especially in diving, pulmonary barotrauma may result in serious complications. The increased risk of pneumothorax associated with changes in atmospheric pressure can be explained by a change in transpulmonary pressure in regions with air trapping, and not in direct connection with the airways, resulting in a higher pressure compared to the atmosphere (Scott et al. 1989; Suarez-Varel et al. 2000; Bense 1984; Bulajich et al. 2005; Haga et al. 2013). Air trapping may be caused by peripheral airway inflammation with a check-valve mechanism resulting in obstruction of the airway (Smit et al. 2004; Cottin et al. 1998). In addition, lung cysts, emphysematous blebs or bullae may predispose to air trapping (Edmonds et al. 1992). Examples of diseases with cystic lesions in the lung are lymphangioleiomyomatosis (LAM) and Birt–Hogg–Dubé syndrome (BHD). BHD is an autosomal dominant condition caused by germline mutations in the *folliculin* (*FLCN*) gene, clinically characterized by skin fibrofolliculomas, pulmonary cysts (recurrent) spontaneous pneumothorax and an increased risk of renal cell cancer. Pneumothorax may be the first and only manifestation of BHD both in isolated and familial cases. Approximately 90 % of BHD patients show multiple lung cysts on standard CT. Lung function generally remains unaffected, demonstrated by normal spirometry and diffusion capacity (Johannesma et al. 2014a).

Detailed analysis of resected lung specimens of 50 patients with BHD containing a total of 229 cysts was performed by Kumasaka and colleagues. Of the cysts, 50 % was located in the subpleural area and less than 5 % abutted bronchioles (Kumasaka et al. 2014). These findings imply that almost all lung cysts in BHD patients are spaces filled with air without a direct connection to airways and therefore pressure changes outside the body will not rapidly affect the amount of air within the cyst. This situation of trapped air is comparable to a “bag of chips” during air travel (Baumann 2009).

According to Boyle’s law the inverse relationship between pressure and volume for gas in a closed system at a constant temperature will result in an increased size of the cyst during flying and subsequently risk of rupture of the cyst wall. During diving the pressure increases and the cyst will decrease in size reaching pressure equilibrium during the dive. However, after reaching pressure equilibrium during the period under water, the cyst will re-expand to its original size during ascent to the surface and the risk of rupture of the cyst wall is comparable to the situation during air travel.

Few data are available regarding the prevalence of pneumothorax associated with air travel in patients with the interstitial cystic lung disease lymphangioleiomyomatosis (LAM). Two studies retrospectively reviewed the occurrence of spontaneous pneumothorax related to air travel in a group of patients with LAM and found an overall incidence of pneumothorax between 1.1 and 2.2 per 100 flights (Taveira-DaSilva et al. 2009; Pollock-BarZiv et al. 2007).

These findings raise the important clinical question whether BHD patients are as well prone to develop a spontaneous pneumothorax during air travel or after diving. As there is no clear definition of “air travel related pneumothorax” we evaluated the prevalence of spontaneous pneumothorax and adverse events in a period of 1 month both after air travel and after diving.

## Methods

### Study population

We retrospectively approached patients diagnosed in our center with BHD, confirmed by *FLCN* mutation analysis. A survey including a letter summarising the study was sent to the last known address to collect information on demographic data, time since BHD diagnosis, medical history, use of cigarettes and inhaled soft drugs, history of spontaneous pneumothorax, number of SP episodes, side of SP, treatment of SP, air travel and diving behaviour within 1 month before the SP episode and adverse effects during/after flying and/or diving (dyspnoea, chest pain, palpitations, anxiety, fatigue, nausea, dizziness, haemoptysis, headache, blue hands, shackles and/or light headedness). To increase the response rate, a second mailing was sent to non-responders after 6 weeks. The number of flights and the number of diving episodes was divided in none, 1–5 times, 6–10, 11–20 or more than 20. When a patient had a history of more than 20 flights, we noted a mean of 25 flights. Air travel was divided in continental and intercontinental. Depth during diving was categorized in 0–3, 3–10, more than 10 m depth. Written consent was obtained from all subjects.

Medical records of the responders were collected and reviewed for radiological evidence (chest X-ray and/or thoracic CT) of spontaneous pneumothorax. In addition information regarding type of treatment of the (recurrent) spontaneous pneumothorax was collected. Radiological studies for the presence of pneumothorax and lung cysts were reviewed by a radiologist and pulmonologist. The study was approved by the ethics committee of the VU University Medical Center.

## Results

### Demographics

In total 158 (83.2 %) patients completed and returned the questionnaire. Of this group, 9.5 % was active smoker, 42.5 % former smoker (mean: 19 pack years) and 7.6 % had a history of inhaling drugs. In total 145 BHD patients reported having traveled by airplane at least once. We defined a flight as a single flight including one ascent and one descent. A total of 1582 single flights in Europe (mean 10.9 flights) and 946 single intercontinental flights (mean 6.5 flights) were reported. Fifty-four patients (34.2 %) had ever dived, all for recreational purposes. The majority of this group had dived between 1 and 5 times at a depth between 0 and 3 m (56.3 %). Depth was categorized in 0–3 m (87 %), 3–10 m (48.1 %), >10 m (14.8 %).

### Thoracic CT in BHD patients with a history of spontaneous pneumothorax after air travel or diving

A thoracic CT was available for nine patients with a history of SP after air travel (N = 8) or diving (N = 1). The number of cysts on standard thoracic CT varied between 1 and 140, with a mean of 57.2 cysts. The majority of cysts was located in the basal parts of the lung equally divided subpleural and parenchymal (Table 2). Whether the number of lung cysts is significantly higher in this patients group, we scored the thoracic CT’s of 42 patients with BHD and a history of air travel and/or diving, but without a history of (recurrent) pneumothorax. The number of cysts on thoracic CT varied between 2 and 33, with a mean of 19 cysts. The number of cysts in patients BHD and a history of pneumothorax was significantly higher compared to the group of patients with BHD but without a history of pneumothorax (p < 0.008).

### Adverse events during air travel and diving

Complaints reported during air travel by BHD patients (N = 145), included shortness of breath (4.1 %), chest pain (6.2 %), palpitations (2.8 %), anxiety (9.7 %), abnormal fatigue (3.4 %), nausea (4.1 %), dizziness (0.7 %), abnormal headache (3.4 %), abnormal chills (1.4 %) and lightheadedness (4.8 %). These subjective symptoms were reported in 30 patients (20.7 %) during flight.

In total 54 patients had “ever” dived, all recreational. Depth was categorized in 0–3 m (87 %), 3–10 m (48.1 %), >10 m (14.8 %). Subjective symptoms were reported by ten patients (18.5 %) during diving: shortness of breath (11.1 %), anxiety (3.7 %), dizziness (1.9 %), abnormal fatigue (1.9 %), abnormal chills (1.9 %), and hemoptysis (1.9 %).

### Spontaneous pneumothorax after air travel

Sixty-one of 145 BHD patients (42.1 %) had a history of both SP and air travel, with a mean of 2.48 episodes of SP (range 1–10). Twenty-four patients (39.3 %) had a history of (separate) episodes of spontaneous pneumothorax on both sides. Thirteen of 145 BHD patients (9.0 %) developed a SP < 1 month after air traveling. In total 35 of 145 BHD patients (24.1 %) had ever travelled intercontinental. Two patients developed a SP two times within 1 month after air travel. One patient developed a SP three times. For five patients this was the first episode of SP. The time interval between flight and radiographic diagnoses of spontaneous pneumothorax are summarized in Table 1. The diagnosis of pneumothorax was confirmed in all patients with chest X-ray, additional thoracic CT was performed in five patients. Retrospectively a thoracic CT was available in eight patients (Table 2). Although these patients did not undergo radiographic imaging of the chest prior to their flight, we assume that these patients developed a pneumothorax during air travel as these patients had no subjective symptoms of pneumothorax before their flight. We calculated a pneumothorax risk of 0.63 % per flight.

**Table 1:** Characteristics of 13 BHD patients with (recurrent) SP after air travel

Patient number	Destination prior to SP	Number of flights in Europe	Number of flights Intercontinental	Recurrent episode of SP?	Episode number of SP	Type of imaging diagnosis SP	Time interval
1	Unknown	>20	>20	Yes	2	X thorax	<48 h
2	Europe	>20	11–20	Yes	3	X	<48 h
3	Europe	>20	1–5	Yes	4	X thorax +CT	10–15 days
4	Europe	>20	>20	No	1	X thorax	5–10 days
5	Intercontinental	>20	>20	No	1	X thorax	<48 h
5	Intercontinental	>20	>20	Yes	4	X thorax +CT	<48 h
6	Intercontinental	>20	>20	Yes	2	X thorax	20–25 days
7	Europe	6–10	0	Yes	10	X thorax	<48 h
8	Europe	6–10	1–5	No	1	X thorax	<48 h
9	Europe	>20	0	Yes	2	X thorax +CT	<48 h
10	Europe	>20	6–10	Yes	2	X thorax	15–20 days
11	Europe	>20	>20	Yes	2	X thorax +CT	20–25 days
12	Intercontinental	>20	>20	No	1	X thorax	15–20 days
13	Europe	>20	>20	No	1	X thorax	5–10 days
13	Intercontinental	>20	>20	Yes	2	X thorax	<48 h
13	Intercontinental	>20	>20	Yes	3	X thorax +CT	<48 h

**Table 2:** Imaging and treatment characteristics of 13 BHD patients with (recurrent) SP after air travel

Patient number	Thoracic CT Available	Number of cysts	Prior SP	Prior pleurodesis	Treatment SP after air travel	*FLCN* mutation
1	Yes	5	Yes	Yes	Pleurodesis	c.610_611delinsTA
2	Yes	1	Yes	No	Pleurodesis	c.499C>T
3	n/a	n/a	Yes	No	Pleurectomy/pleurodesis	c.250-?_1740+?del (del exon 5–14)
4	Yes	99	No	No	Tube thoracostomy	c.1285dupC
5	Yes	140	No	No	Tube thoracostomy	c.1408_1418del; p.Gly470fs
5	Yes	140	Yes	Yes	Pleurodesis	c.1408_1418del; p.Gly470fs
6	Yes	4	Yes	No	Pleurodesis	c.619-1G>A
7	n/a	n/a	Yes	No	Tube thoracostomy	c.1539-2A>G
8	Yes	74	No	No	Pleurodesis	c.1539-2A>G
9	n/a	n/a	Yes	No	Pleural rubbing	c.610_611delinsTA
10	Yes	19	Yes	Yes	Pleurodesis	c.610_611delinsTA
11	n/a	n/a	Yes	No	Pleural rubbing	c.1065_1066delGCinsTA
12	n/a	n/a	No	No	Tube thoracostomy	c.610_611delinsTA
13	Yes	99	No	No	Tube thoracostomy	c.1740dupC
13	Yes	99	Yes	No	Apical pleurectomy	c.1740dupC
13	Yes	99	Yes	No	Total pleurectomy and pleurodesis	c.1740dupC

*n/a* not available

### Spontaneous pneumothorax after diving

Two patients out of 54 that had ever dived (3.7 %) developed a SP < 1 month after diving, both after diving at depth between 3 and 10 m. Although these patients did not undergo radiographic imaging of the chest prior to their diving session, we assume that these patients developed a pneumothorax during ascending to the surface, as both patients had no subjective symptoms of pneumothorax before the diving session. The first patient dived between 6 and 10 times at a depth of 3–10 m. After a diving session of 30 min, this patient developed his first pneumothorax within 48 h. The second patient developed her third episode of pneumothorax after her first diving session of maximum 30 min at a depth of 3–10 m, within 48 h (Table 3). Thoracic CT was available in one patient. Both patients were treated with a VATS procedure with surgical pleurodesis (Table 4). We calculated a pneumothorax risk of 0.33 % per episode of session.

**Table 3:** Characteristics of 2 BHD patients with (recurrent) SP after diving

Patient number	Total episodes of diving^a^	Duration of diving prior to SP (min)	Type of imaging diagnosis SP	Episode number of SP	Depth of diving prior to SP (m)	Time interval (h)
1	6–10	30	X thorax	1	3–10	<48
2	1–5	30	X thorax	3	3–10	<48

^a^All episodes at 3–10 m depth

**Table 4:** Imaging and treatment characteristics of 2 BHD patients with (recurrent) SP after diving

Patient number	Thoracic CT Available	Number of cysts	Prior SP	Prior pleurodesis	Treatment SP after air travel	*FLCN* mutation
1	n/a	n/a	No	No	Pleurodesis	IVS9+6 C>T and IVS8+36G>A
2	Yes	74	Yes	Tube thoracostomy	Pleurodesis	c.1539-2A>G

*n/a* not available

## Discussion

To date, our study is the largest air travel and diving survey of patients diagnosed with BHD. In this retrospective study we analyzed the self-reported history of air travel and diving of 158 patients diagnosed with BHD. In total 13/145 (9.0 %) and 2/54 (3.7 %) patients developed a spontaneous pneumothorax within 1 month after air travel or diving respectively. In total 30/145 (20.1 %) and 10/54 (18.5 %) of BHD patients experienced one or more adverse events during air travel and diving respectively. We calculated a pneumothorax risk of 0.63 % per flight and a pneumothorax risk of 0.33 % per diving session.

The literature regarding the risk for pneumothorax related to diving in patients with lung cysts is extremely limited. However, several case reports suggest a relationship between a bulla and an increased risk for pneumothorax during ascent. So far, a standardised documentation of in-flight medical and surgical emergencies (IMEs) has not been established. Recommendations by several speciality society guidelines and literature reviews addressing time to travel from pneumothorax diagnosis and or treatment vary widely and show some discrepancies, which is probably due to the limited number of studies on this subject. The British Thoracic Society (BTS) guideline recommends diving to be permanently avoided after an episode of spontaneous pneumothorax unless the patient has undergone bilateral surgical pleurectomy and the lung function and postoperative thoracic CT are normal (MacDuff et al. 2010). Recommended time to travel from diagnosis and/or treatment for spontaneous pneumothorax varies between no time period noted up to 21 days after radiographic resolution. Currently, there are no guidelines for patients with cystic lung diseases. We show here that the incidence of pneumothorax in BHD associated with flying and diving might be higher than in the general population and therefore recommendations with respect to air travel and diving for patients with BHD have to be established. Pneumothorax occurring in-flight appears to be rare in the general population. Coker and colleagues described the results on 500 patients with a variety of lung diseases traveling by air. No in-flight emergencies, including pneumothoraces, were reported (Coker et al. 2007). Sand and colleagues identified 10.189 cases of in-flight medical emergencies in a retrospective study of two European airlines; no cases of pneumothorax were documented (Sand et al. 2009). A recent evaluation among 11.920 patients by Peterson and colleagues, showed also no pneumothorax as in-flight emergency. Between 1969 and 2012 a total of 38 episodes of pneumothorax during air travel are described in literature (Peterson et al. 2012). The majority of patients had the cystic lung disease LAM as underlying disease (Hu et al. 2014).

Whether patients diagnosed with BHD are at an increased risk for developing spontaneous pneumothorax associated to air travel or diving is unknown. So far only little data concerning BHD and air travel are available. Hoshika and colleagues surveyed a small population with BHD syndrome (N = 48). None of them had experienced air travel related pneumothorax, although the length of the pneumothorax free period after air travel was not specified (Hoshika et al. 2012). Hu and colleagues reported an overview of 12 reports of in-flight pneumothoraces and associated outcomes but did not specify the disease characteristics of these patients. In only one study the episode of pneumothorax during the flight was fatal (Tiemensma et al. 2010).

LAM, like BHD is a pulmonary disease associated with lung cysts. So far two studies including LAM patients reported the incidence of pneumothorax during air travel. The first study found an in-flight pneumothorax risk of 2.2 % among 308 LAM patients and an estimated pneumothorax risk for LAM patients of 4 % per woman flying. The second study among 449 LAM patients reported an incidence of 1.1 pneumothoraces per 100 flights and 2.9 pneumothoraces per 100 patients. This is a higher incidence than we found in our study. An explanation could be that BHD patients have less cysts in their lungs, as they only appear under the carina.

One may question whether a pneumothorax occurring days or weeks after the event with considerable atmospheric pressure change should be considered as related to that event. The size of the connection between airway and pleural cavity is likely to determine how fast a pneumothorax will increase in size and it may therefore take days to weeks before causing symptoms. To determine whether a pneumothorax indeed occurred during or directly after air travel, imaging before and directly after air travel would be required. Our choice to make an inventory of SP within 1 month after air travel was arbitrary and based on the assumption that these episodes may be related to flying.

Based on histological evaluation of the lung cysts in BHD patients this appears to be plausible. The location of the cysts in the periphery of the lungs, at least 50 % is located subpleurally and the lack of a direct connection with the intrapulmonary airways supports this theory (Medvetz et al. 2012; Postmus et al. 2014; Johannesma et al. 2014b). Based on these characteristics it is unlikely that rupture of a cyst will lead to a considerable flow of air into the pleural cavity. On the contrary, if a subpleural cyst ruptures the overlying visceral pleura has to rupture as well and resulting of the release of a very small amount of air into the pleural cavity. Larger amounts of air can only get there through damage of surrounding parenchyma and/or airways in the periphery adjacent to the ruptured cyst. Ultimately the size of the connection between airway and pleural cavity determines how fast a pneumothorax will increase in size and it may therefore take days to weeks before this will lead to symptoms.

The more reported association between LAM and pneumothorax after air travel might be related to the disease itself. Cysts in LAM might possibly be caused by air trapping behind an obstruction of the airways (Ryu et al. 2006; Chu et al. 1999).

Rupture of these cysts is therefore more likely to include the airway resulting in a larger connection between airway and pleural cavity than in BHD and therefore leading to symptoms earlier.

So far, a standardised documentation of in-flight medical and surgical emergencies (IMEs) has not been established. Recommendations by several speciality society guidelines and literature reviews addressing time to travel from pneumothorax diagnosis and or treatment vary widely and show some disagreement, which is probably due to a lack of evidence-based support structure. Recommended time to travel from diagnosis and/or treatment for spontaneous pneumothorax varies between no time period noted up to 21 days after radiographic resolution, but is not suitable for patients with cystic lung diseases. We show that the incidence of pneumothorax in BHD, and likely in other cystic lung disease as well, is much higher than in the general population and therefore recommendations with respect to air travel for patients with BHD have to be established.

Based on our current data in this retrospective study, we suggest that patients with BHD might possibly be at an increased risk for pneumothorax while flying. Possibly, it may take several days to weeks before a pneumothorax becomes symptomatic. Patients with BHD should be advised that the presence of any clinical symptoms such as shortness of breath or chest pain, during flying or shortly after air travel might indicate a (small) pneumothorax. This should preclude flying and these persons should immediately undergo appropriate radiologic testing, by chest X-ray or thoracic CT, before being approved for air travel or diving.

In summary, SP in BHD patients after air travel and diving might occur more often than in the general population. We found a pneumothorax risk of 0.63 % per flight and a pneumothorax risk of 0.33 % per diving episode. The majority of patients had a history of more than 20 flights. Although BHD is not mentioned in the BTS and ACCP guideline for pneumothorax after air travel or diving, clinicians should be aware of the possible increased pneumothorax risk in BHD patients. An individualized advice should be given, taking also into account patients’ preferences and needs. Further research is required to address the exact rate of pneumothorax during and directly after air travel. Preferably, a healthy control group is used to address the pneumothorax rate in the general population. Since pneumothorax during diving is reported to be associated with serious complications, and screening for cysts has been suggested in professional divers, we recommend that BHD patients are evaluated and counselled on the potentially associated risk by a physician with experience in diving medicine.

## Abbreviations

ACCP: American College of Chest Physicians
BHD: Birt–Hogg–Dubé syndrome
BTS: British Thoracic Society
CT: computed tomography
FLCN: folliculin
IME’s: in-flight medical and surgical emergencies
LAM: lymphangioleiomyomatosis
SP: spontaneous pneumothorax
